# Hip fracture risk in relation to vitamin D supplementation and serum 25-hydroxyvitamin D levels: a systematic review and meta-analysis of randomised controlled trials and observational studies

**DOI:** 10.1186/1471-2458-10-331

**Published:** 2010-06-11

**Authors:** Jeffrey K C Lai, Robyn M Lucas, Mark S Clements, Andrew W Roddam, Emily Banks

**Affiliations:** 1 National Centre for Epidemiology and Population Health, The Australian National University, Canberra, ACT, 0200, Australia; 2 Cancer Epidemiology Unit, University of Oxford, Richard Doll Building, Roosevelt Drive, Oxford, OX3 7LF, UK

## Abstract

**Background:**

Vitamin D supplementation for fracture prevention is widespread despite conflicting interpretation of relevant randomised controlled trial (RCT) evidence. This study summarises quantitatively the current evidence from RCTs and observational studies regarding vitamin D, parathyroid hormone (PTH) and hip fracture risk.

**Methods:**

We undertook separate meta-analyses of RCTs examining vitamin D supplementation and hip fracture, and observational studies of serum vitamin D status (25-hydroxyvitamin D (25(OH)D) level), PTH and hip fracture. Results from RCTs were combined using the reported hazard ratios/relative risks (RR). Results from case-control studies were combined using the ratio of 25(OH)D and PTH measurements of hip fracture cases compared with controls. Original published studies of vitamin D, PTH and hip fracture were identified through PubMed and Web of Science databases, searches of reference lists and forward citations of key papers.

**Results:**

The seven eligible RCTs identified showed no significant difference in hip fracture risk in those randomised to cholecalciferol or ergocalciferol supplementation versus placebo/control (RR = 1.13[95%CI 0.98-1.29]; 801 cases), with no significant difference between trials of <800 IU/day and ≥800 IU/day. The 17 identified case-control studies found 33% lower serum 25(OH)D levels in cases compared to controls, based on 1903 cases. This difference was significantly greater in studies with population-based compared to hospital-based controls (χ^2^_1 _(heterogeneity) = 51.02, p < 0.001) and significant heterogeneity was present overall (χ^2^_16 _(heterogeneity) = 137.9, p < 0.001). Serum PTH levels in hip fracture cases did not differ significantly from controls, based on ten case-control studies with 905 cases (χ^2^_9 _(heterogeneity) = 149.68, p < 0.001).

**Conclusions:**

Neither higher nor lower dose vitamin D supplementation prevented hip fracture. Randomised and observational data on vitamin D and hip fracture appear to differ. The reason for this is unclear; one possible explanation is uncontrolled confounding in observational studies. Post-fracture PTH levels are unrelated to hip fracture risk.

## Background

Vitamin D supplementation is widely considered to be an important therapy for the prevention of fracture and use for this purpose is both widespread and recommended, with and without calcium [1-3]. Previous meta-analyses of randomised controlled trials found either no significant effect of vitamin D on fracture risk [4,5] or have been interpreted as indicating that vitamin D doses of ≥700-800 IU/day [6] or "received doses" of ≥400 IU/day [7] are required to prevent fracture. Notably, however, key higher dose trials in these latter meta-analyses were trials of placebo versus combined vitamin D plus calcium supplementation [8-11] and calcium supplementation is now known to have an independent protective effect on fracture [12]. This raises questions on the efficacy and necessity of vitamin D supplementation independent of calcium.

Hip fracture is the most serious outcome of osteoporosis and an important and increasing health problem. It is common amongst older individuals and is associated with significant morbidity and mortality. In 2000, there were estimated 1.6 million hip fractures worldwide [13] and mortality in the year following hip fracture is estimated at 20-30% [14]. The social and economic burden of hip fractures worldwide is expected to increase significantly over the next 50 years due to ageing populations, especially within developing countries [15-17]. Identification of interventions that can prevent hip fractures remains a key research priority and vitamin D is an appealing therapy to fulfil this role.

In addition to the randomised data, the opinions and practices of researchers, clinicians and communities are likely to be influenced by factors including observational studies and the longstanding knowledge of the relationship between vitamin D and osteomalacia. In the face of the continuing uncertainty and the need for firm evidence to guide practice, an up to date and broader quantitative examination of the evidence regarding vitamin D and fracture is warranted. Shrier et al suggest that the advantages of examining different levels of evidence by including observational studies with RCTs in meta-analyses may outweigh the disadvantages [18]. This study is designed to be the first summary of the available serological evidence on both vitamin D and PTH in relation to hip fracture, and the first meta-analysis of case-control studies on this topic.

## Methods

### Search Strategy and Eligibility

We applied the inclusion and exclusion criteria below to studies identified through searches of the PubMed and Web of Science databases, additional articles identified from the reference lists of sourced papers, hand searching of relevant journals and forward citations searches of key papers, to include publications up to April 2009.

Studies were included if they were published, English language, original research articles and were one of the following.

a) A randomised controlled trial including a minimum of 100 participants combined across vitamin D treatment (cholecalciferol or ergocalciferol) and control groups, with at least one radiologically confirmed hip fracture in each group; b) A case-control study including a minimum of 50 cases of hip fracture together with specified control participants and post-fracture serum 25(OH)D (the usual blood measure of vitamin D status) and/or PTH levels. Studies must have reported sufficient data to allow the calculation of a mean and standard deviation for serological measurements; or c) A cohort study recording confirmed incident hip fracture and the relationship to serum 25(OH)D levels ascertained from prospectively collected blood samples.

Exclusion criteria included randomised controlled studies that used vitamin D treatment combined with other therapies such that the individual effect of vitamin D could not be established [8-11]; studies that included hip fractures as a component of all fractures but without individual counts; and studies published as abstracts only.

Although only studies from English-language publications were included, PubMed encompasses abstracts from non-English publications. This revealed only one relevant non-English language study [19] and this abstract provided similar results to the other studies that were included.

Data were independently extracted by three reviewers (JL, EB, RL) and discrepant results reconciled through arbitration. Two of the reviewers (EB and RL) are researchers and specialist public health physicians and one (JL) has qualifications in statistics. Adjusted results were used where possible. JL conducted the quality assessment.

### Quality Assessment of included studies

Randomised controlled trials were assessed for quality based upon allocation concealment, blinding of interventions, and the total loss to follow up. Lower quality studies were those considered to be inadequate in any of the above areas (no allocation concealment, no blinding of interventions or >25% loss to follow up) including studies where the description presented was not clear. We investigated heterogeneity between lower and higher quality studies.

For observational studies, the quality assessment was based upon adjustment for confounding (none = 1 point, age and sex = 2 points, age and sex plus other possible confounders = 3 points), the selection of cases and controls over a comparable time period (1 point for same period or season), and the adequate description of patient characteristics (1 point for inclusion of characteristics such as residential status, sunshine exposure, calcium intake and medical history). These broad criteria were taken from the list of most commonly used measures in a review of quality assessments of observational studies [20]. Adjustment for confounding was weighted more heavily as it was considered the best proxy for study quality. Based upon the number of points scored out of a maximum of 5, each study was given a quality rating from 1 (lowest) to 5 (highest). We investigated heterogeneity between lower quality studies (1 and 2) compared to higher quality studies (3, 4 and 5).

### Statistical analysis and presentation of results

For the randomised controlled trials we tabulated the measure of association for hip fracture comparing vitamin D treatment and control groups. Some studies provided the hazard ratio from a Cox Proportional Hazards model. For studies where this was not available, we calculated the relative risk from raw data [21,22]. To check the validity of directly comparing these measures of association, we calculated the relative risk (RR) (from the raw data) for all studies and performed a sensitivity analysis using a standard t-test with unequal variances to compare the results obtained by this method to those using the adjusted hazard ratio. This revealed that the treatment effects using both methods were statistically equal (p = 0.99) and as such the adjusted results were used where possible. Outcomes were analysed on an intention-to-treat basis. We examined heterogeneity of results according to the type and dose of vitamin D used, mode of administration, use of placebo, reported levels of compliance, and according to whether or not participants were community dwelling or institutionalized, using the Cochrane Q statistic [23,24] with the p-value obtained from a chi-squared distribution with *n-1 *degrees of freedom. Overall heterogeneity between individual studies was investigated using the Cochrane Q statistic in addition to the H and I^2 ^statistics with the p-value obtained from a standard normal distribution. The I^2 ^statistic is a transformation of the H statistic and represents 'the proportion of variance attributable to heterogeneity'. Further details on its calculation are outlined in Higgins et al [25]. Cut-offs for heterogeneity were taken at p = 0.05. We tested for publication bias using funnel plots and asymmetry determined using Egger's linear regression approach with the p-value obtained from a t-distribution with *n-2 *degrees of freedom [26].

Most case-control studies reported the mean and standard deviation of serum 25(OH)D levels directly. However, in studies where participants were stratified according to gender or season, we obtained the combined standard deviation from the usual ANOVA sum-of-squares breakdown.

Within case-control studies, 25(OH)D and PTH measurements of hip fracture patients and controls were compared using a ratio estimator of the respective means. To account for the positively skewed distribution common in serological measurements a logarithmic transformation of the data was used to better approximate normality. As most studies reported only the arithmetic mean and standard error, it was necessary to estimate the geometric mean and standard error on the log transformed scale using asymptotic Taylor series approximations. Details of the methodology used are outlined in Higgins et al [27].

Results were summarised across studies using the weighted average of the study-specific log ratios, with each individual weight inversely proportional to its estimated variance. Graphically log ratios are represented as black squares with areas proportional to their weights indicating the amount of statistical information for each particular study. The corresponding confidence interval (CI) is drawn as a line extending from the estimated log ratio.

We decided *a priori *to test for heterogeneity according to the use of population- or hospital- based controls. Hospital-based controls were defined as hospital inpatients or outpatients identified for inclusion in the study either during or as a direct result of diagnostic or medical treatment. All other controls drawn from home-based or housing for the elderly populations were taken as population-based controls. We further tested for heterogeneity due to geographical location, mean age of controls, time between fracture and serum collection, and type of vitamin D assay.

All analyses were undertaken with the R computing package (version 2.5.1; 2007, available at: http://www.cran.r-project.org) [28].

Major results from cohort studies were presented as reported in the original studies. A meta-analysis of estimates was not possible due to the different cut points for serum 25(OH)D from each study.

## Results

### Randomised controlled trials

**Figure 1:**
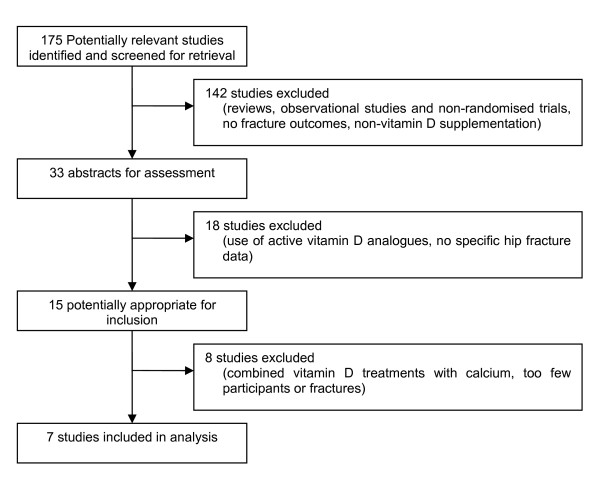
**Study selection of randomised controlled trials**.

A total of seven eligible randomised controlled trials recording hip fractures was identified (see Figure 1) [21,22,29-33]. Six studies were vitamin D versus placebo or "no treatment" trials [22,29-33], and one was a factorial design investigating vitamin D versus placebo and vitamin D and calcium versus calcium alone [21]. The same study was a secondary fracture prevention study that selected participants based upon a previous fracture history. Table 1 summarises the studies including study populations, vitamin D dose and type, mode of administration, change in 25(OH)D levels and the relative risk or hazard ratio reported in the study paper.

**Table 1:** Summary of identified randomised controlled trials

Study first author (country) year	Study population	Mean age (years)	Vitamin D				Control		Hip Fractures	RR/HR
						
			n	Eqv daily dose and type	Mode of admin	25(OH)D Baseline nmol/L	n	25(OH)D Baseline nmol/L	Number Vit D/ Control	
**Vitamin D vs. Placebo/No treatment trials**										

Lips [29] (Netherlands) 1996	Community & limited care residents	80	1291	400 IU	oral	27	1287	26	58/48	HR
				D_3_						1.18 (0.81-1.71)

Meyer [30] (Norway) 2002	Nursing home residents	84.7	569	400 IU	oral	47	575	51	50/47	HR
				D_3_						1.09 (0.73-1.63)

Trivedi [31] (UK) 2003	Community Doctors' and GP registry	74.8	1345	822 IU	oral	NA	1341	53.4	21/24	HR
				D_3_						0.85 (0.47-1.53)

Grant [21] (UK) 2005	Community residents with previous fracture	77	1343	800 IU	oral	38.0	1332	38.0	47/41	RR
				D_3_						1.14 (0.75-1.72)

Law [32] (UK) 2006	Residential care and nursing home residents	85	1762	1100 IU	oral	59	1955	NA	24/20	RR
				D_2_						1.36 (0.80-2.34)

Lyons [22] (UK) 2007	Residential, nursing homes and sheltered housing residents	84	1725	822 IU	oral	NA	1715	NA	112/104	RR
				D_2_						1.07 (0.83-1.39)

Smith [33] (UK) 2007	Community residents	79.1	4727	822 IU	Intra-musc. (IM)	141.25	4713	141.25	66/44	HR
				D_2_						1.49 (1.02-2.18)

**Vitamin D and Calcium vs. Calcium trials**										

Grant [21] (UK) 2005	Community residents with previous fracture	77	1306	800 IU D_3 _+ 1000 mg Calcium(daily)	oral	38.0	1311	38.0	46/49	RR
										0.94 (0.63-1.40)

Eqv Equivalent

RR Relative risk

HR Hazard Ratio

IU International Units

**Figure 2:**
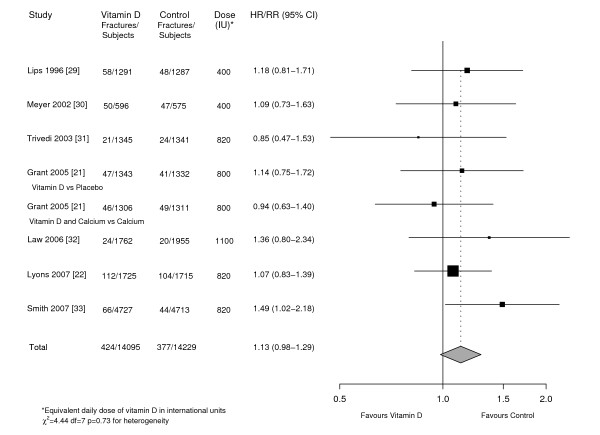
**HR/RR of hip fracture in randomised trials**. Hazard Ratio/Relative Risk of hip fracture in individuals randomised to vitamin D supplementation compared to placebo/control in eligible randomised trials

The pooled analysis of these studies (Figure 2) included 424 and 377 hip fractures in the vitamin D and control groups respectively. The weighted RR was 1.13 (95%CI, 0.98-1.29). There was no evidence of heterogeneity (χ^2^_7 _= 4.44, p = 0.73 and H = 1, I^2 ^= 0%, p = 1), including in relation to geographical location. All studies reported adequate allocation concealment; one study did not use placebo and did not adequately blind intervention [32] and two studies reported a loss to follow up over the course of the study of >25% [21,22]. Further, one study did not report loss to follow up figures [33]. Performing a sub-group analysis comparing these studies with the higher quality studies showed no evidence of heterogeneity (χ^2^_1 _= 0, p = 1). No significant variations were found between results of studies randomising participants to: <800 IU/day 1.14 (95%CI, 0.86-1.49) or ≥800 IU/day 1.12 (95%CI, 0.96-1.32); vitamin D_2 _1.21 (95%CI, 0.99-1.48) or vitamin D_3 _1.06 (95%CI, 0.88-1.28); oral 1.08 (95%CI, 0.94-1.25) or intramuscular injections 1.49 (95%CI, 1.02-2.18); placebo 1.11 (95%CI, 0.97-1.28) or no placebo 1.36 (95%CI, 0.80-2.34); reported levels of non-compliance (≤15% 1.26 [95%CI, 1.03-1.55] vs >15% 1.03 [95%CI, 0.86-1.24]); or between nursing home 1.11 (95%CI, 0.91-1.36) and community residents 1.14 (95%CI, 0.95-1.37). There was no evidence of publication bias.

### Case-control studies

#### Vitamin D status

A total of 17 eligible studies of hip fracture and serum 25(OH)D levels were identified, including a total of 1903 hip fracture cases and 1953 control participants [34-50]. Eleven studies specified the time at which blood samples were obtained and all but one gave the assay method used to determine 25(OH)D concentration. The collection of sera ranged from the point of admission to hospital up to a maximum mean of 35 days post-fracture. The reported average age in all studies for both cases and controls ranged from 69 [48] to 84 years [49].

Of the 17 case-control studies, nine were population-based [34-36,38-42,44] and eight were hospital-based [37,43,45-50]. There was a strong relationship between control type and year of publication. Eight of the nine studies published in 1992 or earlier were population-based case-control studies whilst seven of the remaining eight studies published in 1995 or later were hospital-based case-control studies.

**Figure 3:**
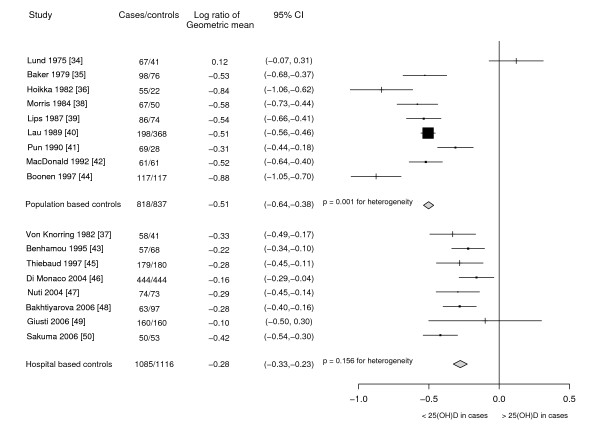
**25(OH)D in case-control studies**. Ratio of serum 25(OH)D levels in hip fracture patients compared to controls in eligible case-control studies.

Table 2 shows characteristics of the studies including control populations, mean age, time of serum collection, assay technique, and geometric mean serum 25(OH)D and parathyroid hormone (PTH) levels. Figure 3 presents a plot of the studies, stratified according to the source of controls. There was no evidence of heterogeneity in relation to geographical location, mean age of controls, time between fracture and serum collection or type of vitamin D assay.

**Table 2:** Characteristics of Hip fracture case-control studies

Study first author (location) year	Cases			Controls		Participant details
		
	n	Mean Age (SD) years 25(OH)D (SD) nmol/L PTH (SD) pmol/L	Time sera collected (25(OH)D assay method)	n	Mean Age (SD) years 25(OH)D (SD) nmol/L PTH (SD) pmol/L	
**Population based controls**						

Lund [34]	67	NR	On admission	41	NR	*Cases: *Consecutive patients
(Denmark) 1975		65.0 (45.0) NR	(CPB)		55.0 (32.5) NR	*Controls: *Elderly participants without malabsorption, hepatic or renal disease

Baker [35]	98	80.2 (7.3)	-	76	79.4 (7.2)	*Cases: *Consecutive admissions of Caucasian females
(UK) 1979		34.5 (24.5) NR			55.8 (33.8) NR	*Controls: *Home-based age and sex matched selected from electoral register

Hoikka [36]	55	76.0	-	22	71.0	*Cases: *Patients admitted from Sept 1978- July 1979
(Finland) 1982		26.6 (18.5) NR	(HPLC)		56.4 (28.2) NR	*Controls*: Healthy age, sex and season matched

Morris [38]	67	77.9	-	50	71.5	*Cases: *Female hip fracture patients operated on by author
(Australia) 1984		39.2 (21.3) NR	(CPB)		67.6 (30.4) NR	*Controls: *Home-based, ambulant elderly females

Lips [39]	86	73.1 (11.5)	-	74	75.6 (4.2)	*Cases: *Hip fracture patients
(Netherlands) 1987		20.4 (11.5)	(CPB after HPLC)		32.9 (13.6)	*Controls*: Healthy, independent volunteers living in apartment house for elderly
		0.11 (0.05)*			0.12 (0.05)*	

Lau [40]	198	NR	On day of admission	368	NR	*Cases: *Consecutive patients
(Hong Kong) 1989		45.5 (16.4) NR	(CPB)		73.8 (21.0) NR	*Controls: *Sheltered housing residents

Pun [41]	69	78.1 (10.2)	Within 12 h admission	28	71.2 (6.4)	*Cases: *Female hip fracture patients
(Hong Kong) 1990		43.7 (22.4) NR	(CPB)		54.6 (13.1) NR	*Controls: *Healthy female participants over 60 y

MacDonald [42]	61	78.9 (10.7)	Within 24 h admission pre-surgery	61	78.6 (6.0)	*Cases: *Unselected Chinese female patients admitted to orthopaedic ward
(Hong Kong) 1992		45.8 (22.0)	(CPB)		72.5 (21.5)	*Controls: *Hostel for elderly, no fracture history
		4.59 (2.27)			4.04 (2.24)	

Boonen [44]	117	79.2 (8.9)	Within 18 h fracture pre-surgery	117	77.7 (5.4)	*Cases: *Consecutive female patients
(Belgium) 1997		25.3 (22.0)	(CPB)		53.8 (33.3)	*Controls: *Female, age matched, from general practices
		5.14 (4.66)			1.70 (1.14)	

**Community based controls**						

Von Knorring [37]	58	77.0 (9.3)	Prior to surgery	41	78.0 (8.4)	*Cases: *Patients admitted during 2 month periods in summer, winter and early spring
(Finland) 1982		32.4 (17.0)	(CPB)		44.7 (22.5)	*Controls: *Age and sex matched non-orthopaedic outpatients or minor surgery patients
		0.45 (0.24)*			0.35 (0.24)*	

Benhamou [43]	57	83.9 (5.9)	Within 24 h admission	68	82.5 (5.0)	*Cases: *Consecutively admitted patients
(France) 1995		30.9 (12.2)	(RIA)		38.4 (14.7)	*Controls: *Patients in geriatric or rheumatology unit with no bone disease or fracture history
		6.34 (4.65)			4.74 (2.21)	

Thiebaud [45]	179	80.2 (9.6)	5-18 post fracture	180	79.9 (9.6)	*Cases: *Consecutive admissions of original hip fracture caused by fall from standing height or less
(France) 1997		23.5 (20.3)	(Radio-receptor)		30.6 (25.4)	*Controls: *Age and sex matched emergency patients with no fracture history
		3.32 (2.77)			4.62 (3.38)	

Di Monaco [46]	444	79.7 (8.6)	During hosp mean 35 days post fracture	444	75.5 (5.7)	*Cases: *Caucasian patients sustaining original hip fracture
(Italy) 2004		21.2 (19.5)	(immuno-enzymatic)		24.4 (21.7)	*Controls: *Home-based elderly over 65 y referred for first osteodensiometry, no fracture history
		6.02 (4.56)			5.28 (2.32)	

Nuti [47]	74	77.4 (9.3)	Within 24 h fracture	73	71.2 (6.1)	*Cases: *Self-sufficient, community-living female patients with adequate sunlight admitted between May-Dec 1999
(Italy) 2004		83.5 (55.0)	(CPB)		107.3 (60.0)	*Controls: *Metabolic disease unit outpatients with osteoporosis, no evident osteoporotic fractures admitted between Jul-Nov 1999. Otherwise as per cases
		4.02 (1.79)			3.86 (1.43)	

Bakhtiyarova [48]	63	68.8 (9.5)	Within 3 days admission	97	70.2 (8.3)	*Cases: *Low trauma fracture patients
(Russia) 2006		22.4 (11.4)	(CPB)		28.1 (10.1)	*Controls: *Patients of ophthalmology unit with no fracture
		3.7 (3.3)			4.9 (2.8)	

Giusti [49]	160	84.0 (6.3)	Within 24 h admission (RIA)	160	82.0 (7.6)	*Cases: *Sampled patients admitted to Orthogeriatric unit between Nov 2004-Mar 2005
(Italy) 2006		9.4 (11.6)			9.2 (9.1)	*Controls: *Age, sex and place matched Acute Care unit patients admitted for non-bone reasons
		8.95 (7.79)			9.16 (8.00)	

Sakuma [50]	50	82.6 (8.7)	On admission	53	77.2 (5.3)	*Cases: *Sado Island residents admitted to hospital from Jan-Dec 2004
(Japan) 2006		44.5 (19.4)	(ELISA)		64.5 (18.5)	*Controls: *Orthopaedic patients with no fracture admitted between Jul-Dec 2004
		4.75 (2.34)			3.31 (1.93)	

CPB: competitive protein binding

HPLC: high profile liquid chromatography

RIA: radioimmunoassay

ELISA: enzyme linked immunoassay

NR: not reported

* non-standardised units

Studies using population-based controls included 818 hip fracture cases and 837 controls. There was significant heterogeneity (χ^2^_8 _= 76.28, p < 0.001 and H = 3.09, I^2 ^= 89.5%, p < 0.001) largely driven by three outlying studies [34,36,44], however the direction of the difference was consistent in all but one study. The weighted average of log ratios showed around 40% (log ratio -0.51) lower serum vitamin D levels in hip fracture cases compared with population-based controls.

The hospital-based case-control studies included 1085 hip fracture cases and 1116 controls. In seven of the eight studies, cases had significantly lower serum 25(OH)D concentrations than controls. The combined log ratio showed around 24% (log ratio -0.28) lower serum vitamin D levels in hip fracture patients compared to hospital-based controls with no significant heterogeneity (χ^2^_7 _= 10.62, p = 0.156 and H = 1.23, I^2 ^= 34.1%, p = 0.319). The summary results for population-based and hospital-based case-control studies differed significantly (χ^2^_1 _= 51.02, p < 0.001).

Overall, in 15 of the 17 case-control studies hip fracture patients had significantly lower 25(OH)D levels than controls. A total combined estimate showed around 33% (log ratio -0.40) lower 25(OH)D level in cases compared to controls, although significant heterogeneity existed between studies (χ^2^_16 _= 137.9, p < 0.001 and H = 2.94, I^2 ^= 88.4%, p < 0.001). This broad summary should therefore be regarded with caution.

Seven studies were identified as lower quality studies with a rating of either 1 [34,40] or 2 [38,41,42,46,48]. Comparing these studies with the higher quality studies with ratings 3 [37,43,47,50], 4 [35,36,39,45], or 5 [44,49], there were no signs of heterogeneity (χ^2^_1 _= 0.14, p = 0.71). There was no evidence of publication bias.

#### Parathyroid hormone

**Figure 4:**
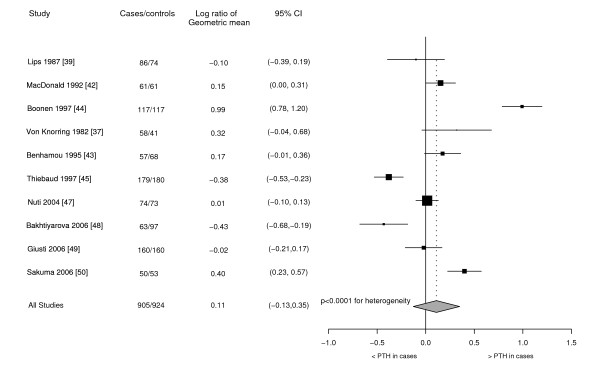
**PTH in case-control studies**. Ratio of serum PTH levels in hip fracture patients compared to controls in eligible case-control studies.

Ten eligible case-control studies of PTH and hip fracture were identified. These were essentially a subset of studies examining 25(OH)D [37,39,42-45,47-50]. Figure 4 presents the studies identified, including 905 hip fracture cases and 924 controls from three population-based case-control and seven hospital-based case-control studies. Two of the studies showed significantly higher PTH levels in hip fracture patients compared to controls [44,50] while two studies showed relationships in the opposite direction with significantly lower PTH levels in the hip fracture patients compared to controls [45,48]. The other six studies showed no significant difference between case and control groups. These results were not related to the time between fracture and serum collection. There was no evidence of publication bias.

There was significant and substantial heterogeneity between study results within both the subgroups of population-based case-control and hospital-based case-control studies. After combining the estimates, there was no significant difference between the PTH levels in cases and controls (difference in cases compared to controls 12%, log ratio 0.11) although substantial heterogeneity was evident overall (χ^2^_9 _= 149.68, p < 0.001, and H = 4.08, I^2 ^= 94%, p < 0.001) and this summary result should therefore be interpreted with caution. Two studies were identified as lower quality studies with a rating of 2 [42,48]. There were no signs of heterogeneity between studies with lower, compared to higher (3 [37,43,47,50], 4 [39,45], or 5 [44,49]), quality ratings (χ^2^_1 _= 2.52, p = 0.112).

### Cohort studies

Table 3 shows the three identified cohort studies investigating 25(OH)D levels and hip fracture [51-53]. Two studies used nested case-control designs. One of these studies did not find a significant association between serum 25(OH)D and hip fracture using a cut-off point of 47.5 nmol/L [51]. Another study containing 400 hip fractures found a dose-related increase in hip fracture risk for lower serum 25(OH)D levels (OR = 1.33 [95%CI, 1.06-1.68] for each 25 nmol/l decrease) [52], while the third study concluded that there was a significantly reduced risk of hip fracture in those with 25(OH)D levels greater than or equal to 62.5 nmol/L compared to levels below this (RR = 0.64 [95%CI, 0.48-0.89]) [53]. For comparability with the previous studies, the reciprocal of this value is RR = 1.56 (95%CI, 1.12-2.08). Despite the differences in the cutoff points in serum 25(OH)D levels used in these analyses, the results of the cohort studies are essentially consistent with one another and qualitatively similar to those of the case-control studies, with overlapping confidence intervals and generally elevated risks of hip fracture in those with lower 25(OH)D levels. It should be noted that in the one study that permitted the comparison of mean 25(OH)D levels in cases and controls, the difference appeared less extreme than that seen in the case-control studies, although 25(OH)D levels were still statistically different (cases 55.95 nmol/L [SD 20.28] vs. controls 59.60 nmol/L [SD 18.20], p = 0.007).

**Table 3:** Characteristics of cohort studies

Study First author (country) year	n	Follow up time	Study population Mean age years	Hip Fractures	Findings
Study of Osteoporotic Fractures	9704	Up to 5.9 years	Postmenopausal women	332	< 47.5 nmol/L vs. ≥47.5 nmol/L 25(OH)D
Cummings [51] (USA) 1998			73 years (using randomly selected cases and controls)		RR Hip fracture 1.2 (0.7, 1.9)

WHI-OS	39793	Up to 9.3 years	Postmenopausal women	400	Each 25 nmol/L decrease in 25(OH)D R Hip fracture 1.33 (1.06, 1.68)
Cauley [52] (USA) 2008		(median 7.1 years)	71 years		≤47.5 nmol/L vs. ≥70.7 nmol/L OR Hip fracture 1.71 (1.05, 2.79)

NHANES III	1917	Mean 6.7 y	≥65 y Caucasian adults	156	≥62.5 nmol/L vs. <62.5 nmol/L 25(OH)D
Looker [53] (USA) 2008			73 years		RR Hip fracture 0.64 (0.46, 0.89)

## Discussion

This meta-analysis shows no significant difference in the risk of hip fracture between individuals randomised to receive either vitamin D supplements or placebo/control. In particular, no significant benefit for hip fracture was shown in trials randomising participants to receive high dose vitamin D (i.e. doses of 800 IU per day or greater). In apparent contrast, case-control studies show substantially and significantly lower serum 25(OH)D levels in persons with hip fractures compared to controls.

### Strengths and Weaknesses

A strength of this meta-analysis is the presentation of a comprehensive collection of randomised controlled trials, case-control studies and cohort studies, allowing a broader comparison of published results than previously considered. The effect of vitamin D supplementation was separated from that of other medications in the RCT data. Clearly defined methods were used in the selection of the studies and analysis of the results to ensure validity and consistency. Furthermore, this represents the first summary of serological evidence on both vitamin D and PTH in relation to hip fracture, and the first meta-analysis of case-control studies on this topic. By placing the evidence of the case-control studies alongside the results of the randomised controlled trials we highlight the complexity of the problem and the difficulty in drawing adequate conclusions.

This study is constrained by the detail and quality of published data of the respective studies included. Many of the case-control studies were relatively small, contained few hip fractures and reported highly variable serum measurements. Individual participant data were rarely published especially in larger studies, limiting a more detailed analysis of patient characteristics. The substantial heterogeneity in the results of the population-based case control studies of 25(OH)D and hip fracture, and the studies of PTH levels mean that the summary results should be considered as providing a broad indication of the overall direction of study findings, rather than a precise estimate of the combined study results. Despite some studies being of lower quality, investigations revealed no heterogeneity between results of higher and lower quality studies, both for randomised controlled trials and case-control studies.

Other potential limitations may include measurement error in 25(OH)D levels from poor assay standardisation [54], misclassification bias in hip fracture status or publication bias from the inclusion of smaller studies.

### Other studies

There have been several meta-analyses of randomised controlled trials published on this topic. A Cochrane review showed similar results for hip fracture risk to the present study with a summary RR of 1.15 (95%CI, 0.99-1.33), in individuals randomised to receive vitamin D compared to placebo or control [5]. A pooled analysis of individual level randomised controlled trial data showed a borderline statistically non-significant decrease in hip fracture risk from vitamin D + calcium supplementation and no reduction in risk from vitamin D supplementation alone [55]. However, these reviews did not consider results from observational studies or the role of PTH as we have done here. Shrier et al suggest that the advantages of including observational studies with RCTs in meta-analyses may outweigh the disadvantages [18]. By considering the evidence in its entirety we are able to offer plausible hypotheses for the apparent null effect seen in RCTs.

Several explanations have been previously proposed for the null Randomised Controlled Trial results. A previous meta-analysis of the relationship between vitamin D supplementation and overall fracture risk stratified by dosage concluded that supplementation of 700-800 IU/d is necessary to reduce non-vertebral fractures [6]. This was followed up most recently by the same authors with another meta-analysis supporting the same conclusion of a dose-dependence [7]. However, these studies did not assess the impact of vitamin D alone but rather included combined treatments with calcium, and calcium alone has been shown to have a significant impact on fracture risk [12]. The latter meta-analysis of calcium treatment reported that the addition of trials of vitamin D + calcium to calcium-only trial results did not change the treatment effect significantly. The RR (95%CI) for fractures at all sites for calcium-only supplementation compared to placebo was 0.90 (95%CI, 0.80-1.00), which became 0.87 (95%CI, 0.77-0.97) when calcium + vitamin D versus placebo trial results were added; these RR did not differ significantly from one another (p = 0.63). This highlights the critical question of whether vitamin D supplementation is itself effective, either with or without calcium supplementation.

### Explaining the inconsistent randomised and observational results

Where randomised and observational evidence are inconsistent, it is generally most appropriate to place more weight on the randomised evidence for clinical decision making. Although the reason for the apparent difference between the randomised and observational evidence on vitamin D and hip fracture is not known, there are a number of possible explanations, the most obvious being that the results of the observational studies may be affected by uncontrolled confounding. The risk of hip fracture is increased by many factors such as advancing age, lack of physical activity, low body mass index, smoking, co-morbidities and frailty. Many of these factors are also likely to reduce exposure to sunlight and therefore vitamin D levels. Since only six of the case-control studies summarised here accounted for age and none adjusted for current illness or disability, it seems likely that the results of the observational studies may be subject to residual confounding. This is supported by our finding of a greater difference in 25(OH)D levels in case-control studies using population rather than hospital controls. Previous studies suggest hospital patients and nursing home residents may have frailty patterns more comparable to hip fracture patients and have lower 25(OH)D levels compared to independent elderly participants [56] who are likely more ambulant and healthier. One of the cohort studies did attempt to adjust for frailty and physical functioning [52] and these factors were found to attenuate the association between low serum vitamin D status and hip fracture risk, although it remained marginally statistically significant. Further similar investigations are necessary to better quantify the likely impact of such confounding and may help explain these conflicting results.

A second explanation is that vitamin D is indeed beneficial and the randomised controlled trials may not have been able to detect this effect. There may have been inadequate power in the trials because of limited follow up and the relatively small number of recorded hip fractures. However the results of a Cochrane review of all fracture sites (summary RR = 1.01 [95%CI, 0.93-1.09]), including more events with greater statistical power, and vertebral fractures only (RR = 0.90 [95%CI, 0.42-1.92]) were similar to those reported here, in individuals randomised to receive vitamin D compared to placebo or control [5]. Performing the analysis on an intention-to-treat basis is consistent with recommendations [57] although it may obscure the effect of poor adherence to treatment or follow up that may reduce the observed treatment effect. However, not all studies reported detailed information on adherence rates and of those that did, four estimated the proportion of adherent participants greater than 75% [22,29-31], and only one study reported lower adherence to treatment of around 50% [21]. This latter study reported a similar treatment effect compared to other studies where there was better adherence.

Another proposed explanation for the different findings of RCTs and observational studies, in particular the lack of a significant treatment effect in the RCTs, is that the doses of vitamin D used in many of the studies may have been too low to achieve hip fracture reduction. Some studies suggest that a minimum supplementation of 700-800 IU/d is necessary to reduce non-vertebral fractures [6]. However as already noted, the treatment effect of trials within this dosage range were not found to differ significantly from those in lower dose trials in the present meta-analysis. Furthermore, the doses of vitamin D supplementation used in both high and low dose randomised controlled trials resulted in increases in serum 25(OH)D levels that were similar to, or in excess of, the differences between cases and controls in the observational data [21,29,30,32,33]. This suggests that the apparent discrepancies between the observational and randomised data are unlikely to be explained by dose alone; it remains possible, though currently unsupported, that very high doses of vitamin D could prevent fracture.

Finally, it is possible that some other factor, such as exposure to sunlight or genotypic factors, independently reduces hip fracture risk and is related to increased serum 25(OH)D levels, but is not simulated by vitamin D supplementation.

### PTH and hip fracture

There was no significant difference between PTH levels in hip fracture cases and controls. Low 25(OH)D levels lead to a small decrease in serum 1,25(OH)_2_D and calcium absorption that in turn stimulates the secretion of PTH to maintain adequate 1,25(OH)_2_D production and calcium homeostasis [56]. This secondary hyperparathyroidism leads to increased bone turnover, bone loss and possibly increased hip fracture risk. It might be expected then that hip fracture patients compared to controls would have elevated PTH measurements consistent with lower 25(OH)D levels. This was not evident although there was significant heterogeneity between studies.

The heterogeneity was not attributable to the timing of sera collection or stratification by population-based or hospital-based controls. However, PTH can vary significantly over a short period and the impact on serum measurements from fracture and subsequent trauma is not well understood. One study showed elevated PTH levels immediately after hip fracture that fell significantly two weeks later [58], while others have reported serum levels increasing [59] or remaining stable [60] during hip fracture recovery. Comparatively, 25(OH)D has a much longer half-life in circulation and studies have consistently showed no changes in 25(OH)D levels from post-fracture through to recovery [58,61].

### Implications and future research

Despite the null findings from the randomised controlled trials, vitamin D is widely recommended and used with the aim of preventing fracture. Current guidelines for bone health support both vitamin D supplementation only, starting from as little as 400 IU/day for individuals who may be sunlight deprived [1], and combined vitamin D and calcium supplementation, as part of broader osteoporosis treatment [3]. However despite current practice, the question still remains of whether vitamin D itself is actually effective. There is insufficient evidence at present to support widespread vitamin D supplementation for fracture prevention. In particular, lack of consistency within the epidemiological evidence highlights the possible impact of bias and confounding, as well as uncertainties on dosage and therapy requirements.

The issue of confounding between vitamin D, physical activity and comorbidity has implications beyond the investigation of vitamin D and fracture. Physical activity, and related body mass index, influences the risk of a wide range of conditions and frailty and co-morbidity have profound impacts on survival. Recently, low vitamin D has been suggested as a risk factor for a number of conditions, including prostate cancer [62], breast cancer [63,64], type 1 and 2 diabetes [65], hypertension [66], multiple sclerosis [67,68] and is speculated to reduce survival from melanoma [69]. These types of observations are potentially affected by similar issues to those outlined here and high quality evidence, ensuring that potential confounding factors have been properly taken into account, is required before definitive conclusions can be reached regarding the effects of vitamin D. It should also be borne in mind that a number of previous observational studies showed reduced rates of cancer and other diseases in individuals taking supplements such as beta-carotene, vitamin A and vitamin E [70]. Subsequent randomised controlled trials revealed that these supplements did not significantly prevent disease [71] and in the case of beta-carotene led to an increased risk of cancer and cardiovascular disease in smokers [72,73].

The way forward for fracture should focus upon strengthening the evidence, informed by the results of previous studies. Specifically, observational studies with tighter control for confounding are likely to be informative. Randomised controlled trials using larger vitamin D doses and combined vitamin D and calcium therapies (versus calcium alone), with appropriate control groups may also contribute to our understanding. Studies should account appropriately for factors such as age, physical activity, functional capacity and appropriate lifestyle factors when assessing the relationship between vitamin D status and hip fracture. Genetic studies examining factors relating to 25(OH)D levels may also provide useful insights for future research [74-76].

## Conclusions

A summary of the best available evidence shows that neither higher nor lower dose vitamin D supplementation prevents hip fracture. Randomised and observational findings on vitamin D and hip fracture appear to differ. The reason for this is unclear; one possible explanation is uncontrolled confounding in observational studies.

## Competing interests

The authors declare that they have no competing interests.

## Authors' contributions

JL participated in the Study conception and design, Acquisition of data, Analysis and Interpretation of data, Statistical analysis, Drafting the manuscript and Critical revision of the manuscript for important intellectual content. RL participated in the Acquisition of data, Analysis and Interpretation of data, Drafting the manuscript and Critical revision of the manuscript for important intellectual content. MC participated in the Analysis and Interpretation of data, Statistical analysis and Critical revision of the manuscript for important intellectual content. AR participated in the Analysis and Interpretation of data, Statistical analysis and Critical revision of the manuscript for important intellectual content. EB participated in the Study conception and design, Acquisition of data, Analysis and Interpretation of data, Drafting the manuscript and Critical revision of the manuscript for important intellectual content. All authors approved the final version of the article.

## Pre-publication history

The pre-publication history for this paper can be accessed here:

http://www.biomedcentral.com/1471-2458/10/331/prepub
